# Racial and ethnic patterns and differences in health care expenditures among Medicare beneficiaries with and without cognitive deficits or Alzheimer’s disease and related dementias

**DOI:** 10.1186/s12877-020-01888-y

**Published:** 2020-11-18

**Authors:** Sungchul Park, Jie Chen

**Affiliations:** 1 Department of Health Management and Policy, Dornsife School of Public Health, Drexel University, 3215 Market Street, Philadelphia, PA USA; 2 Department of Health Policy and Management, School of Public Health, University of Maryland, 4200 Valley Drive, College Park, MD USA

**Keywords:** Alzheimer’s disease and related dementias, Cognitive limitations, Racial and ethnic disparity, Health care expenditure, out-of-pocket expenditure

## Abstract

**Background:**

Numerous studies have documented racial and ethnic differences in the prevalence and incidence of Alzheimer’s disease and related dementias (ADRD). Less is known, however, about racial and ethnic differences in health care expenditures among older adults at risk for ADRD (cognitive deficits without ADRD) or with ADRD. In particular, there is limited evidence that racial and ethnic differences in health care expenditures change over the trajectory of ADRD or differ by types of service.

**Methods:**

We examined racial and ethnic patterns and differences in health care expenditures (total health care expenditures, out-of-pocket expenditures, and six service-specific expenditures) among Medicare beneficiaries without cognitive deficits, those with cognitive deficits without ADRD, and those with ADRD. Using the 1996–2017 Medical Expenditure Panel Survey, we performed multivariable regression models to estimate expenditure differences among racial and ethnic groups without cognitive deficits, those with cognitive deficits without ADRD, and those with ADRD. Models accounted for survey weights and adjusted for various demographic, socioeconomic, and health characteristics.

**Results:**

Black, Asians, and Latinos without cognitive deficits had lower total health care expenditures than whites without cognitive deficits ($10,236, $9497, $9597, and $11,541, respectively). There were no racial and ethnic differences in total health care expenditures among those with cognitive deficits without ADRD and those with ADRD. Across all three groups, however, Blacks, Asians, and Latinos consistently had lower out-of-pocket expenditures than whites (except for Asians with cognitive deficits without ADRD). Furthermore, service-specific health care expenditures varied by racial and ethnic groups.

**Conclusions:**

Our study did not find significant racial and ethnic differences in total health care expenditures among Medicare beneficiaries with cognitive deficits and/or ADRD. However, we documented significant differences in out-of-pocket expenditures and service-specific expenditures. We speculated that the differences may be attributable to racial and ethnic differences in access to care and/or preferences based on family structure and cultural/economic factors. Particularly, heterogeneous patterns of service-specific expenditures by racial and ethnic groups underscore the importance of future research in identifying determinants leading to variations in service-specific expenditures among racial and ethnic groups.

## Background

The prevalence of Alzheimer’s disease and related dementias (ADRD) is a growing crisis in the United States (US) that is estimated to increase substantially over the next several decades. In 2010, approximately 4.5 million Americans had been diagnosed with ADRD [1]. The number of Americans with ADRD is projected to be 13.8 million in 2050 [1]. Furthermore, evidence suggests that care for ADRD generates substantial health care costs [2–5]. Mean per-person costs for Americans with ADRD were $49,126 in 2016, more than triple the average $15,550 per-person costs for Americans without ADRD [5]. Aggregate costs for Americans with ADRD are expected to increase from $172 billion in 2010 to $1.1 trillion in 2050 [5]. Such a dramatic increase in the costs of ADRD will lead to a substantial burden on the US health care system.

In particular, this has critical implications for the Medicare program, which is a federally funded government health insurance program for people age 65 and older and those with disabilities in the US. The Medicare program covers a variety of acute and post-acute care services, including inpatient hospital stays, skilled nursing care, hospice, and some home health care. It also covers certain physicians and other health care providers’ services, outpatient care, medical supplies, and some preventive services. The Medicare program, however, has limited coverage of long-term care. Most Medicare beneficiaries with ADRD pay out-of-pocket or rely on other supplemental insurance programs or Medicaid to cover long-term care.

Numerous studies have documented racial and ethnic differences in the prevalence and incidence of ADRD. Specifically, compared to non-Latino whites (whites), non-Latino Blacks (Blacks) are approximately two times more likely to have ADRD [6, 7] and Latinos are approximately 1.5 times more likely to have ADRD [6, 8, 9]. Recent research found that differences among racial and ethnic groups in the prevalence of ADRD decreased between 2000 and 2012 [10]. However, the prevalence rates of ADRD were still found to be higher among Blacks and Latinos than among whites (19.3, 16.3, and 7.4% for Blacks, Latinos, and whites, respectively). Incidence rates of ADRD were also higher among Blacks and Latinos than among whites (13.8, 12.2, and 10.3% for Blacks, Latinos, and whites, respectively) [11].

While existing studies have examined health care expenditures among Medicare beneficiaries with mild cognitive limitation and/or ADRD, evidence of racial and ethnic disparities is relatively lacking [12, 13]. One study used Medicare fee-for-service claims data for 2014 and found that compared to whites with ADRD, Blacks, Latinos, and “others” with ADRD had higher Medicare expenditures ($27,315, $26,280, $21,649, and $20,199 for blacks, others, Latinos, and whites, respectively) [11]. Higher expenditures among racial and ethnic minority groups with ADRD might be attributable to limited access to care in the early stages of ADRD, which could lead to delays in treatment and diagnosis and exacerbate disease progression at later stages.

It is also important to understand patterns of type-specific health care expenditures; focusing only on total health care expenditures might lead to overlooking mechanisms that contribute to health care expenditures among members of racial and ethnic minority groups with ADRD. This is more likely to be relevant to patients with ADRD because cultural preferences can affect the optimal clinical setting for individuals with ADRD and their families. Prior research found that caregivers of Black patients were less satisfied with hospital discharge planning than caregivers of white patients were, and that caregivers of Black patients used formal home care more than caregivers of white patients did [14, 15]. Furthermore, there were substantial racial and ethnic differences in the number of individuals who chose to be admitted to nursing homes; usage of nursing homes was particularly low among Latinos [16].

However, it is worth noting that these findings may also be attributable to structural barriers. Additionally, choice of care setting for patients with ADRD is critical because evidence suggests that these patients experience inefficient care delivery and high health care utilization. For example, a significant factor driving high health care utilization among those with ADRD is transitions to high-cost settings such as an inpatient setting or skilled nursing facility [17–19]; some of these transitions have been shown to be unnecessary or preventable [20–23]. This suggests that higher expenditures among patients with ADRD might result from inefficient use of care.

To address this gap, we examined racial and ethnic patterns and differences in health care expenditures among Medicare beneficiaries. We estimated such expenditures among Medicare beneficiaries with cognitive deficits without a diagnosis of ADRD and those diagnosed with ADRD. In addition, we examined various types of health care expenditures: total health care expenditures, out-of-pocket (OOP) expenditures, and six service-specific expenditures.

## Methods

### Data and sample

We used data from the 1996–2017 Medical Expenditure Panel Survey (MEPS). MEPS is a nationally representative survey of the US non-institutionalized civilian population. MEPS annually collects information on respondents’ demographic and socioeconomic characteristics, health status, and health care expenditures. Two datasets from MEPS were included in our analyses: the full year consolidated data files and the medical conditions files. The full year consolidated data file contains information on demographic and socioeconomic characteristics and health care expenditures. The medical conditions file provides information on medical conditions associated with medical events from respondents as verbatim text and coded by professional coders using the *International Classification of Diseases, Ninth Revision, Clinical Modification* (ICD-9-CM) or the *International Classification of Diseases, Tenth Revision, Clinical Modification* (ICD-10-CM). Using an individual unique identifier, we linked the full year consolidated data file to the medical conditions file for each year.

We included Medicare beneficiaries (aged 65 and above) who were non-Latino white, non-Latino Black, non-Latino Asian, or Latino. Then, we identified the following three populations: [1] those who reported as not having cognitive deficits, [2] those who reported as having cognitive deficits without a diagnosis of ADRD, and [3] those diagnosed with ADRD. MEPS measured cognitive deficits based on the household respondent’s answers for the individuals in the sample. Cognitive deficits were assessed through the following three questions: whether the individual experienced confusion or memory loss, whether the individual had problems making decisions, or whether the individual required supervision for their own safety. Cognitive deficits were defined as having any of the three conditions. It is worth noting that there are other tests to assess cognitive impairment or functioning and most of them use a multidimensional measure. Thus, our measure of cognitive deficits is likely to capture a wide range of cognitive impairment. ADRD cases were identified through three-digit ICD-9-CM diagnostic codes (290, 294, 331, or 797) [3, 13] or three-digit ICD-10 diagnostic codes (F01, F03, G30, and G31) [24]. Because the transition to ICD-10 diagnostic codes was implemented in October 2015, we used the ICD-9-CM diagnostic codes for the data between 1997 and 2014 and the ICD-10 diagnostic codes for the data between 2016 and 2017. We used the ICD-9-CM and ICD-10 diagnostic codes for the 2015 data.

### Measurements

Our outcomes included eight health care expenditures: [1] total health care expenditures, [2] OOP expenditures, and [3] six service-specific expenditures. Service-specific expenditures included inpatient expenditures, outpatient expenditures, office-based expenditures, emergency room (ER) expenditures, home health expenditures, and prescription drug expenditures. All health care expenditures were adjusted to 2019 dollars using the Personal Consumption Expenditures Price Index for health care [25].

The key independent variables were the participant’s race (white, Black, Asian, or Latino), presence of cognitive deficits or ADRD, and its interaction terms. To control for differences in sample characteristics among racial and ethnic groups, we included the following variables: age (65–69, 70–74, 75–79, 80–84, or ≥ 85 years old); sex; marital status (married or unmarried); education (less than high school degree, high school degree, some college, or more than college degree); family income as a share of the federal poverty level (FPL; 0–99%, 100–124%, 125–199%, 200–399%, or ≥ 400%); family size (one, two, three, or more than four); health insurance in addition to Medicare (private health insurance or Medicare and Medicaid dual coverage); area of residence (Northeast, Midwest, South, or West); medical conditions (myocardial infarction, congestive heart failure, diabetes, hypertension, diabetes, renal disease, cancer, and psychiatric disorder); limitations at school, work, or housework; functional limitations; and a proxy response to an interview (proxy response or self-response).

### Statistical analysis

We first estimated weighted sample characteristics among racial and ethnic groups without cognitive deficits, those with cognitive deficits without ADRD, and those with ADRD and tested differences using chi-squared tests. Then, we examined unadjusted weighted outcomes and used analysis of variance to examine differences among racial and ethnic groups without cognitive deficits, those with cognitive deficits without ADRD, and those with ADRD. Finally, we performed multivariable regression models to estimate expenditure differences among racial and ethnic groups without cognitive deficits, those with cognitive deficits without ADRD and those with ADRD. Specifically, because we observed that all Medicare beneficiaries had non-zero total health care expenditures, we ran generalized linear models to estimate differences in total health care expenditures. We performed the modified Park test [26] and the Pregibon link test [27] and then chose gamma family and log link. For other types of health care expenditures, there were those with zero expenditures and thus we ran two-part models to handle zero expenditures. Using marginal effects at representative values, we produced findings that can be interpreted as dollar values [28, 29]. Specifically, we estimated the adjusted mean values of the outcomes for each of the racial and ethnic groups without cognitive deficits, those with cognitive deficits without ADRD, and those with ADRD. Then, we conducted post-estimation tests to examine statistical significance in the differences in the adjusted outcomes among racial and ethnic minority groups relative to non-Latino white. All models accounted for survey weights and were adjusted for the variables described above as well as year-fixed effects. All analyses were conducted using Stata 15.

## Results

Our sample consisted of 57,057 Medicare beneficiaries without cognitive deficits (39,767 whites, 7974 Blacks, 2551 Asians, and 6765 Latinos), 10,088 Medicare beneficiaries with cognitive deficits without ADRD (5947 whites, 1933 Blacks, 523 Asians, and 1685 Latinos), and 3420 Medicare beneficiaries with ADRD (2028 whites, 693 Blacks, 120 Asians, and 579 Latinos) (Table 1). There were significant differences in sample characteristics among racial and ethnic groups without cognitive deficits, those with cognitive deficits without ADRD, and those with ADRD. For all populations, Blacks, Asians, and Latinos were more likely than whites to have less than a high school degree, more likely to have a family income lower than 200% of the FPL, more likely to have a family with more than three members, and more likely to have private health insurance or Medicare and Medicaid dual coverage. For those without cognitive deficits, there were differences in health status. However, differences were marginal among racial and ethnic groups for those with cognitive deficits without ADRD and those with ADRD.

**Table 1:** Sample characteristics

	Without cognitive deficits							With cognitive deficits without ADRD							With ADRD						
	NL white (*N* = 39,767)	NL Black (*N* = 7974)	*p* value	NL Asian (*N* = 2551)	***p*** value	Latino (*N* = 6765)	***p*** value	NL white (*N* = 5947)	NL Black (*N* = 1933)	***p*** value	NL Asian (*N* = 523)	***p*** value	Latino (*N* = 1685)	***p*** value	NL white (*N* = 2028)	NL Black (*N* = 693)	***p*** value	NL Asian (*N* = 120)	***p*** value	Latino (*N* = 579)	***p*** value
Age (years)			***		***		***			***		***		***			**				*
65–69	29.54	35.72		34.02		35.65		18.00	24.08		18.60		23.59		5.11	5.92		5.93		5.94	
70–74	26.37	27.42		27.42		28.74		17.54	21.65		20.93		21.46		9.42	13.02		5.93		14.86	
75–79	20.78	18.53		19.70		19.65		19.90	18.70		22.29		22.31		15.04	19.53		19.49		17.66	
80–84	14.15	11.63		11.02		10.11		20.62	16.70		18.41		15.50		25.87	21.15		30.51		27.62	
> = 84	9.16	6.70		7.84		5.84		23.93	18.86		19.77		17.14		44.56	40.38		38.14		33.92	
Female	57.08	63.19	***	56.47		58.29		60.42	66.39	**	60.47		67.96	***	67.34	72.93	**	73.73	*	65.21	
Married	3.19	6.89	***	3.62		5.83	***	4.78	8.96	***	5.04		7.54	**	3.04	5.33		6.78		7.34	***
Education			***		***		***			***		***		***			***		***		***
Less than high school degree	16.18	29.04		30.48		54.23		27.33	41.99		41.67		65.29		27.59	42.75		58.47		67.83	
High school degree	42.73	43.70		25.55		24.95		42.30	37.04		23.45		18.12		43.04	37.57		17.80		14.69	
Some college or more than college degree	36.22	19.95		33.03		12.62		24.51	11.85		21.71		8.09		21.92	9.32		11.02		5.59	
Family income			***		***		***			***		***		***			***		**		***
0–99% FPL	9.92	24.77		12.97		24.68		16.91	37.04		20.35		33.31		17.47	32.69		23.73		41.78	
100–124% FPL	5.33	9.69		6.45		10.23		9.28	13.65		8.53		14.29		10.28	11.69		5.93		13.81	
125–199% FPL	16.13	20.58		14.60		22.08		21.68	22.34		20.35		24.13		23.24	24.41		18.64		20.80	
200–399% FPL	30.69	27.47		28.09		27.77		29.83	19.81		25.58		21.52		26.99	21.89		30.51		19.76	
> = 400% FPL	37.93	17.48		37.88		15.23		22.31	7.17		25.19		6.75		22.03	9.32		21.19		3.85	
Family size			***		***		***			***		***		***			***		*		***
1	32.60	38.19		17.63		26.10		43.97	47.00		31.01		34.29		45.11	38.02		39.83		34.79	
2	57.08	36.65		46.88		43.23		39.60	29.29		34.69		33.80		37.06	28.70		30.51		33.39	
3	5.56	11.72		11.14		12.80		6.76	9.69		11.05		11.73		5.72	12.57		5.93		11.01	
4+	2.42	9.21		19.66		14.76		4.53	8.64		19.57		16.60		3.90	9.91		14.41		13.46	
Additional health insurance			***		***		***			***		***		***			***		**		***
Private insurance	38.12	61.42		60.80		77.57		50.26	77.92		74.61		89.36		51.09	80.77		68.64		91.96	
Medicaid	8.47	24.49		30.04		35.76		20.67	46.79		52.52		60.91		22.33	51.04		54.24		68.18	
US census region			***		***		***			***		***		***			***		***		***
Northeast	18.86	17.25		13.29		14.51		17.66	16.49		12.79		16.05		19.19	14.35		14.41		15.03	
Midwest	26.48	17.64		6.21		5.51		23.98	15.91		6.78		3.65		23.95	18.20		2.54		2.10	
South	34.91	57.90		13.29		42.05		37.53	59.59		10.08		40.49		35.49	57.1		17.80		52.62	
West	19.76	7.21		67.21		37.93		20.82	8.01		70.35		39.82		21.37	10.36		65.25		30.24	
Medical conditions																					
Myocardial infarction	6.15	5.26	***	4.18	***	5.52	*	10.74	11.59		7.75	**	8.88		7.90	11.24	*	9.32		6.12	
Congestive heart failure	4.21	4.02		2.39	***	2.46	***	9.17	8.69		5.04	**	5.59	***	8.96	9.91		6.78		6.12	
Diabetes	17.43	33.18	***	25.47	***	33.66	***	27.61	41.94	***	33.33		44.44	***	20.81	35.80	***	27.12	*	40.21	***
Hypertension	55.24	76.02	***	64.82	***	63.32	***	60.85	78.71	***	70.54		69.12	***	56.76	76.78	***	75.42	**	68.36	***
Renal disease	0.60	1.38	***	0.48		0.53	***	1.69	3.11	*	2.33		1.34		1.16	1.92		0.00		1.05	
Caner	14.86	7.23	***	4.97	***	5.89	***	16.38	8.75	***	3.29	***	6.87	***	12.15	9.17		1.69	**	8.22	*
Mental disease	15.45	8.88	***	5.09	***	13.60	***	36.35	21.18	***	18.99	***	32.77		32.56	21.75	***	16.1	***	31.64	
Any limitations	53.34	53.14		33.59	***	46.34	***	94.23	95.15		90.31	**	93.37		93.52	96.45		94.92		95.45	
Physical limitations	37.24	41.46	***	20.10	***	31.85	***	84.66	87.04		80.04	***	83.77		77.97	81.80		81.36		81.99	
Proxy respondent	98.97	98.56	**	98.17	***	99.17	*	91.44	92.99		93.99		94.71	***	76.66	84.02	**	78.81		89.34	***

*Notes*: *ADRD* Alzheimer’s disease and related dementias, *NL* non-Latino, *FPL* federal poverty level. ^*^ *P* < .05, ^**^ *P* < .01, ^***^ *P* < .001

There were significant differences in unadjusted health care expenditures among racial and ethnic groups without cognitive deficits, those with cognitive deficits without ADRD, and those with ADRD (Table 2). Blacks, Asians, and Latinos without cognitive deficits had significantly lower total health care expenditures than their white counterparts. Asians with cognitive deficits without ADRD had significantly lower expenditures than the equivalent whites. However, no significant differences were detected among Blacks and Latinos with cognitive deficits without ADRD and Blacks, Asians, and Latinos with ADRD. For most OOP expenditures, Blacks, Asians, and Latinos in all groups had significantly lower expenditures than the equivalent whites. With one exception, there was no significant difference in OOP expenditures between whites and Asians with ADRD. For service-specific expenditures, Blacks, Asians, and Latinos without cognitive deficits tended to have lower inpatient, outpatient, office-based, home health, and prescription drug expenditures than the equivalent whites. However, significant differences were detected in a few types of expenditures among racial and ethnic groups with cognitive deficits without ADRD and those with ADRD (home health expenditures among Blacks with cognitive deficits without ADRD, inpatient expenditures among Asians with cognitive deficits without ADRD, outpatient and office-based expenditures among Latinos with cognitive deficits without ADRD, and inpatient and office-based expenditures among Latinos with ADRD).

**Table 2:** Unadjusted expenditures among Medicare beneficiaries with and without cognitive deficits or ADRD by race/ethnicity

	Expenditures ($), Mean (SD)							
	Total	Out-of-pocket	Inpatient	Outpatient	Office-based	ER	Home health	Prescription drug
Without cognitive deficits								
NL white (*N* = 39,767)	11,686 (19001)	1876 (2683)	3696 (13532)	2063 (8685)	5992 (11978)	247 (1604)	317 (2718)	2720 (5323)
NL Black (*N* = 7974)	10,522 (18551)^***^	1217 (3394) ^***^	3370 (12418)	1592 (8706)^**^	4518 (12144)^***^	284 (1410)	734 (4701)***	2720 (5222)
NL Asian (*N* = 2551)	7782 (19638)^***^	1115 (2688)^***^	2164 (16959) ^***^	669 (4733)^***^	3999 (8641)^**^	117 (528)	300 (2696)^**^	2328 (4885)^**^
Latino (*N* = 6765)	9070 (17012)^***^	1032 (2077)^***^	2908 (12711)^**^	947 (4638)^***^	4220 (10430) ^***^	269 (2261)	572 (3989)^***^	2380 (3916)^***^
With cognitive deficits without ADRD								
NL white (*N* = 5947)	21,222 (31181)	2617 (5319)	8464 (23907)	2188 (10782)	7150 (15071)	471 (1637)	2712 (10424)	4165 (5905)
NL Black (*N* = 1933)	21,628 (30511)	1656 (3729)^***^	7871 (20071)	1994 (11807)	7135 (20664)	480 (1653)	4154 (11259)^***^	4124 (8734)
NL Asian (*N* = 523)	15,930 (21125)^**^	1429 (5464)^***^	4525 (12573) ^**^	1039 (5299)	6272 (15183)	424 (1618)	3165 (9368)	3775 (5320)
Latino (*N* = 1685)	19,425 (30296)	1159 (1994)^***^	7437 (23572)	1384 (6383)^*^	5679 (12715)^**^	378 (1377)	3447 (10719)	4119 (5766)
With ADRD								
NL white (*N* = 2028)	21,830 (27119)	4037 (9629)	6089 (17392)	1073 (5054)	5324 (11214)	489 (1349)	6744 (16028)	4476 (5292)
NL Black (*N* = 693)	24,752 (26767)	1897 (4936)^***^	8922 (20413)^**^	1060 (6407)	3983 (8534)^*^	504 (1663)	8250 (13558)	4174 (4893)
NL Asian (*N* = 120)	20,040 (29550)	1903 (6406)	5441 (22930)	147 (691)	3934 (6762)	466 (2729)	6817 (14605)	4377 (5180)
Latino (*N* = 579)	24,318 (31543)	1415 (3371)^***^	6262 (23446)	1149 (5986)	5031 (12812)	659 (2375)	9334 (16572)	4543 (5333)

*Notes*: *ADRD* Alzheimer’s disease and related dementias, *ER* emergency room, *NL* non-Latino

^*^ *P* < .05, ^**^ *P* < .01, ^***^ *P* < .001

Our adjusted analysis showed that Blacks, Asians, and Latinos without cognitive deficits had lower total health care expenditures than whites without cognitive deficits ($10,236 (*P* < .001), $9497 (*P* < .001), $9597 (*P* < .001), and $11,541, respectively), but there were no racial and ethnic differences in total health care expenditures among those with cognitive deficits without ADRD and those with ADRD (Table 3). In all populations, however, Blacks, Asians, and Latinos tended to have lower OOP expenditures than whites (except for Asians with cognitive deficits without ADRD). The magnitude of the differences in OOP expenditures was most pronounced for those with ADRD.

**Table 3:** Adjusted expenditures among Medicare beneficiaries with and without cognitive deficits or ADRD by race/ethnicity

	Expenditures ($), Mean (95% CI)							
	Total	Out-of-pocket	Inpatient	Outpatient	Office-based	ER	Home health	Prescription drug
Without cognitive deficits								
NL white (*N* = 39,767)	11,541 (11,323 to 11,759)	1826 (1796 to 1855)	3634 (3476 to 3792)	2027 (1936 to 2118)	5512 (5388 to 5636)	228 (216 to 241)	362 (330 to 393)	2725 (2665 to 2785)
NL Black (*N* = 7974)	10,236 (9633 to 10,839)^***^	1286 (1226 to 1347)^***^	3388 (2979 to 3796)	1689 (1464 to 1914)^***^	4440 (4166 to 4714)^***^	279 (237 to 321)^*^	624 (523 to 724)^***^	2304 (2204 to 2403)^***^
NL Asian (*N* = 2551)	9497 (8540 to 10,454)^***^	1444 (1278 to 1609)^***^	2968 (2082 to 3853)	941 (695 to 1187)^***^	4386 (3893 to 4879)^***^	133 (101 to 165)^***^	357 (214 to 499)	2678 (2432 to 2924)
Latino (*N* = 6765)	9597 (9054 to 10,139)^***^	1304 (1212 to 1395)^***^	3082 (2643 to 3521)^*^	1396 (1173 to 1618)^***^	4674 (4386 to 4962)^***^	249 (187 to 311)	616 (483 to 749)^***^	2320 (2183 to 2458)^***^
With cognitive deficits without ADRD								
NL white (*N* = 5947)	21,133 (20,134 to 22,132)	2420 (2262 to 2578)	8388 (7627 to 9148)	2064 (1805 to 2324)	6489 (6090 to 6888)	459 (411 to 508)	3075 (2748 to 3402)	4422 (4227 to 4617)
NL Black (*N* = 1933)	21,299 (19,488 to 23,109)	1722 (1494 to 1950)^***^	7303 (6268 to 8339)	2012 (1483 to 2541)	6881 (5805 to 7957)	448 (368 to 529)	4561 (3848 to 5273)^***^	3948 (3422 to 4474)
NL Asian (*N* = 523)	19,090 (15,323 to 22,858)	1793 (991 to 2595)	5114 (3364 to 6863)^***^	863 (559 to 1168)^***^	5824 (4516 to 7132)^***^	443 (262 to 624)	4257 (2299 to 6215)	4264 (3651 to 4878)
Latino (*N* = 1685)	19,277 (17,449 to 21,106)	1356 (1226 to 1487)^***^	7435 (5857 to 9013)	1783 (1327 to 2239)	6232 (5556 to 6907)	360 (268 to 453)	3539 (2869 to 4208)	3669 (3376 to 3962)^***^
With ADRD								
NL white (*N* = 2028)	21,839 (20,221 to 23,458)	3394 (2998 to 3790)	5907 (4918 to 6896)	1046 (853 to 1239)	5129 (4394 to 5864)	465 (392 to 537)	7780 (6814 to 8747)	4987 (4647 to 5328)
NL Black (*N* = 693)	22,982 (20,681 to 25,283)	2140 (1653 to 2627)^***^	7223 (5576 to 8871)	1122 (478 to 1765)	4417 (3578 to 5256)	503 (355 to 652)	9316 (7814 to 10,819)	3899 (3436 to 4361)^***^
NL Asian (*N* = 120)	21,164 (14,103 to 28,226)	1631 (394 to 2868)^**^	5898 (597 to 11,199)	159 (−18 to 336)^***^	4252 (2796 to 5709)	137 (59 to 215)^***^	6427 (3626 to 9227)	4790 (3496 to 6085)
Latino (*N* = 579)	22,832 (19,691 to 25,973)	1896 (1379 to 2414)^***^	5462 (3218 to 7706)	1063 (348 to 1779)	5447 (3972 to 6921)	699 (370 to 1028)	9025 (7356 to 10,695)	4303 (3582 to 5024)

*Notes*: *ADRD* Alzheimer’s disease and related dementias, *ER* emergency room, *NL* non-Latino

We performed multivariable regression models to estimate expenditure differences among racial and ethnic groups without cognitive deficits, those with cognitive deficits without ADRD and those with ADRD. Using marginal effects at representative values, we estimated the adjusted mean values of the outcomes for each of the racial and ethnic group without cognitive deficits, those with cognitive deficits without ADRD, and those with ADRD. To control for differences in sample characteristics among racial and ethnic groups, we adjusted for age, sex, marital status, education, family income as a share of the federal poverty level, family size, health insurance in addition to Medicare, area of residence, medical conditions, limitations at school, work, or housework, functional limitations, and a proxy response to an interview. Then, we conducted post-estimation tests to examine statistical significance in the differences in the adjusted outcomes among racial and ethnic minority groups relative to non-Latino white

^*^ *P* < .05, ^**^ *P* < .01, ^***^ *P* < .001

Our adjusted analysis also showed that service-specific health care expenditures varied by racial and ethnic groups. Compared to whites without cognitive deficits, the Blacks had lower $338 outpatient expenditures (*P* < .001), $1072 office-based expenditures (*P* < .001), and $422 prescription drug expenditures (*P* < .05), but higher $50 ER expenditures and $262 home health expenditures (*P* < .05). Compared to whites without cognitive deficits, the Asians had lower $1086 outpatient expenditures (*P* < .001), $1126 office-based expenditures (*P* < .001), and $95 ER expenditures (*P* < .001). Compared to whites without cognitive deficits, the equivalent Latinos had lower $522 inpatient expenditures (*P* < .05), $631 outpatient expenditures (*P* < .001), $838 office-based expenditures (*P* < .001), and $405 prescription drug expenditures (*P* < .001), but had higher $21 ER expenditures (*P* < .001). Compared to whites with cognitive deficits without ADRD, the equivalent Blacks had higher $1486 home health expenditures (*P* < .001), the equivalent Asians had lower $1261 outpatient expenditures (*P* < .001) and $665 office-based expenditures (*P* < .001), and the equivalent Latinos had lower $754 prescription drug expenditures (*P* < .001). Compared to whites with ADRD, the equivalent Blacks had lower $1089 prescription drug expenditures (*P* < .001) and the equivalent Asians had lower $887 outpatient expenditures (*P* < .001) and $328 ER expenditures (*P* < .001). There were no differences in service-specific health care expenditures between whites and Latinos with ADRD.

## Discussion

It has been consistently shown that racial and ethnic minority groups tended to have lower health care expenditures than whites, partly due to limited access to care. Consistent with prior literature, our study found that there were significant differences in total health care expenditures among racial and ethnic groups without cognitive deficits. However, racial and ethnic differences in total health care expenditures were insignificant among those with cognitive deficit limitations without ADRD and those with ADRD. These findings may suggest that overall access to care and treatment are relatively equitable across racial and ethnic groups among Medicare beneficiaries with cognitive deficits or ADRD.

Our study makes several key contributions to the literature. First, we used data that collects information on race and ethnicity via population survey. Prior research has instead relied on the Medicare claims data. A common concern about the claims data is a lack of in-depth measures of socioeconomic factors that may influence the health care expenditures and racial and ethnic disparities. Using the nationally representative survey data enables us to account for comprehensive measures of demographic and socioeconomic factors. Hence, our findings should be more robust and more accurately predict the racial and ethnic disparities in the amount and pattern of health care expenditures. In addition, we examined racial and ethnic disparities along the trajectory of ADRD (i.e., among Medicare beneficiaries without cognitive limitation, those with cognitive limitation without ADRD, and those with ADRD, respectively). Finally, we investigated racial and ethnic differences in health care expenditures across different settings. Our findings can inform the patterns and preferences of health care utilization across racial and ethnic minority groups.

It is worth noting that our study showed the discrepancies between unadjusted summary of expenditures and predicted expenditures after adjusting for individuals’ demographic and socioeconomic characteristics. Our unadjusted analysis showed that Asians with cognitive deficits without ADRD had lower total health care expenditures than other racial and ethnic groups. However, this was not observed among Asians with ADRD. This indicates that Asians with cognitive deficits but no ADRD may not receive timely health care services, possibly leading to late detection and diagnosis of ADRD. This explanation is likely plausible because Asians are more likely to lack a usual source of care relative to whites [30, 31], leading to relatively lower health care utilization [32], especially for primary care, and preventive services [33]. However, a significant difference was not detected in our adjusted analysis, probably due to a small sample size. Also, our unadjusted analysis showed that consistent with findings from previous studies [11, 34, 35], Blacks and Latinos with ADRD had higher total health care expenditures than white counterparts. However, significant differences were not observed after adjusting for demographic and socioeconomic status and health status. This phenomenon was found in incidence [36], but our findings confirmed that a similar result was observed in health care expenditures. This implies that higher expenditures among Blacks and Latinos with ADRD may partly account for lower socioeconomic status and/or poorer health status.

On the other hand, Blacks, Asians, and Latinos had significantly lower OOP expenditures than whites in both populations of those with cognitive deficits without ADRD and those with ADRD. Lower OOP expenditures among the racial and ethnic minority groups are likely to be attributable to differences in insurance coverage. As shown in our study, Blacks, Asians, and Latinos were more likely to have additional insurance coverage such as Medicaid or private health insurance than whites. However, our findings should not be simply interpreted as indicating that the racial and ethnic minority groups have lower financial burden than whites. This is because insurance premiums were not included in estimating OOP expenditures in this study. This may lead to underestimation of OOP expenditures, especially for the racial and ethnic minority groups who were more likely to have private insurance coverage. Moreover, the racial and ethnic minority groups may fear high costs of care, and thus have delayed or forgone care, especially for high-cost services that are less likely to be covered by insurance. Indeed, Latinos and Asians were shown to experience more delayed or forgone care than whites [37]. Finally, the racial and ethnic minority groups may replace high-costs services with informal care by family caregivers. One study found that Latinos and Asians were more likely to use informal home care and less likely to use formal care compared to whites [38]. This could be feasible because of relatively large family sizes commonly attributed to racial and ethnic minority groups.

Our findings showed that service-specific expenditures varied by racial and ethnic groups, but similar trends were observed in both populations. Blacks and Latinos had higher home health expenditures than whites in both populations. This may be attributable to the fact that they prefer home health care due to the presence of family members who can provide informal care [39] or cultural reasons [40]. However, Blacks and Latinos had lower prescription drug expenditures than whites. This is likely to be explained by less contact with physicians, possibly resulting in fewer prescriptions being written [41]. Research found that Blacks and Latinos were more likely to have mental health visits to primary care providers rather than to specialists, leading to fewer prescriptions for psychotropics [42]. On the other hand, Asians with cognitive deficits without ADRD had lower inpatient and outpatient expenditures than the equivalent whites. This may raise concern of delayed detection or diagnosis of ADRD, as diagnostic services for disease detection are usually provided in inpatient or outpatient settings [11]. However, Asians with ADRD had lower outpatient and ER expenditures than the equivalent whites. This may indicate that Asians manage health better as research showed that ADRD patients had rehospitalizations or ER visits mainly due to poor care management such as injuries from falls [43].

Our findings should be interpreted with caution and additional research is warranted to improve our understanding on patterns and differences in health care expenditures among racial and ethnic groups with cognitive deficits or ADRD. Although we did not find significant racial and ethnic differences in total health care expenditures among those with cognitive deficits without ADRD and those with ADRD, this does not necessarily mean that racial and ethnic minority groups have equal access to care because there is ample evidence showing racial and ethnic disparities in cognitive level and ADRD risk [44] and reporting cognitive deficits [45]. Also, heterogeneous patterns of service-specific expenditures by racial and ethnic groups underscore the importance of future research in identifying determinants leading to variations in service-specific expenditures among racial and ethnic groups. It is important to examine whether racial and ethnic differences in service-specific expenditures are attributable to care access or care preference. Identifying sources of the expenditure differences may help better understand underlying mechanisms associated with patterns of health care access and utilization among racial and ethnic groups. If the expenditure differences are mainly driven by limited access to service-specific care, partly due to cultural and health literacy barriers, this suggests a need to develop interventions tailored to meet the needs of racial and ethnic minority groups with cognitive deficits or ADRD.

Our study has several limitations. First, our measure of cognitive deficits was self-reported and thus is subjective to reporting bias. Self-reported cases of cognitive deficits cannot be validated through clinical assessment. Evidence suggests racial and ethnic differences in reporting cognitive deficits [45], indicating that health care expenditures for racial and ethnic minority groups with cognitive deficits without ADRD may be under-estimated. However, this impact might be minimal because our measure of cognitive deficits captures a wide range of cognitive impairment, including very mild and mid cognitive deficits. Also, MEPS surveys the civilian non-institutionalized US population, and thus our estimates did not account for patterns of health care expenditures for the civilian institutionalized US population. Similarly, MEPS does not include health care expenditures for services from skilled nursing facility. As racial and ethnic minority groups were shown to have lower expenditures for services from skilled nursing facility than whites, this is unlikely to reverse our findings. Furthermore, MEPS provides limited information on ADRD severity, and thus we could not completely control for this factor. We controlled for a range of demographic and socioeconomic characteristics, but we could not adjust for all other potential confounding factors. Moreover, the observed prevalence of ADRD may be inaccurate because we were limited to 3-digit ICD-9-CM or ICD-10-CM codes. Additionally, there are some concerns about the potential impacts of the transition from ICD-9-CM codes to ICD-10-CM codes in 2015. Prior research has found mixed results on changes in prevalence [46–48], but there is limited evidence on ADRD. However, this is unlikely to affect our findings because a relatively small proportion of our sample is influenced by the transition. Finally, our findings should be interpreted with caution as we did not examine whether whites have appropriate health care expenditures. Thus, we cannot rule out the possibility that whites may overutilize health care.

## Conclusions

Our study documented that there were significant differences in total health care expenditures among racial and ethnic groups without cognitive deficits, but no significant differences were detected in total health care expenditures among racial and ethnic groups with cognitive deficits without ADRD and those with ADRD. However, there were substantial differences in OOP expenditures and service-specific expenditures among racial and ethnic groups with cognitive deficits without ADRD and those with ADRD. These findings have implications for future research. First, this work emphasizes that service-specific expenditures varied by racial and ethnic groups. Second, heterogeneous patterns of service-specific expenditures by racial and ethnic groups underscore the importance of future research in identifying determinants leading to variations in service-specific expenditures among racial and ethnic groups.

## Data Availability

The datasets analyzed during the current study are available at https://meps.ahrq.gov/mepsweb/.

## Abbreviations

ADRD: Alzheimer’s disease and related dementias
US: United States
OOP: out-of-pocket
MEPS: Medical Expenditure Panel Survey
ICD-9-CM: *International Classification of Diseases, Ninth Revision, Clinical Modification*
ICD-10-CM: *International Classification of Diseases, Tenth Revision, Clinical Modification*
ER: emergency room
FPL: federal poverty level

## References

[1] Hebert LE, Scherr PA, Bienias JL, Bennett DA, Evans DA (2003). Alzheimer disease in the US population: prevalence estimates using the 2000 census. Arch Neurol.

[2] Park S, White L, Fishman P, Larson EB, Coe NB (2020). Health care utilization, care satisfaction, and health status for Medicare advantage and traditional Medicare beneficiaries with and without Alzheimer disease and related dementias. JAMA Netw Open.

[3] White L, Fishman P, Basu A, Crane PK, Larson EB, Coe NB (2019). Medicare expenditures attributable to dementia. Health Serv Res..

[4] Fishman P, White L, Park S, Larson E, Coe NB. The incremental costs of dementia to a Medicare advantage plan: evidence from the ACT study. Medical Care. Forthcoming.

[5] Hurd MD, Martorell P, Delavande A, Mullen KJ, Langa KM (2013). Monetary costs of dementia in the United States. N Engl J Med.

[6] Gurland BJ, Wilder DE, Lantigua R, Stern Y, Chen J, Killeffer EH (1999). Rates of dementia in three ethnoracial groups. Int J Geriatr Psychiatry.

[7] Potter GG, Plassman BL, Burke JR, Kabeto MU, Langa KM, Llewellyn DJ (2009). Cognitive performance and informant reports in the diagnosis of cognitive impairment and dementia in African Americans and whites. Alzheimers Dement.

[8] Haan MN, Mungas DM, Gonzalez HM, Ortiz TA, Acharya A, Jagust WJ (2003). Prevalence of dementia in older latinos: the influence of type 2 diabetes mellitus, stroke and genetic factors. J Am Geriatr Soc.

[9] Samper-Ternent R, Kuo YF, Ray LA, Ottenbacher KJ, Markides KS, Al SS (2012). Prevalence of health conditions and predictors of mortality in oldest old Mexican Americans and non-Hispanic whites. J Am Med Dir Assoc.

[10] Chen C, Zissimopoulos JM (2018). Racial and ethnic differences in trends in dementia prevalence and risk factors in the United States. Alzheimers Dement (N Y).

[11] Alzheimer's Association (2016). 2016 Alzheimer's disease facts and figures. Alzheimers Dement.

[12] Gaugler JE, Hovater M, Roth DL, Johnston JA, Kane RL, Sarsour K (2013). Analysis of cognitive, functional, health service use, and cost trajectories prior to and following memory loss. J Gerontol B Psychol Sci Soc Sci.

[13] Lin PJ, Zhong Y, Fillit HM, Chen E, Neumann PJ (2016). Medicare expenditures of individuals with Alzheimer's disease and related dementias or mild cognitive impairment before and after diagnosis. J Am Geriatr Soc.

[14] Connell CM, Gibson GD (1997). Racial, ethnic, and cultural differences in dementia caregiving: review and analysis. Gerontologist..

[15] Janevic MR, Connell CM (2001). Racial, ethnic, and cultural differences in the dementia caregiving experience: recent findings. Gerontologist..

[16] Feng Z, Fennell ML, Tyler DA, Clark M, Mor V (2011). The care span: growth of racial and ethnic minorities in US nursing homes driven by demographics and possible disparities in options. Health Aff (Millwood)..

[17] Khandker RK, Black CM, Xie L, Kariburyo MF, Ambegaonkar BM, Baser O (2018). Analysis of episodes of care in Medicare beneficiaries newly diagnosed with Alzheimer's disease. J Am Geriatr Soc.

[18] Teno JM, Gozalo PL, Bynum JP, Leland NE, Miller SC, Morden NE (2013). Change in end-of-life care for Medicare beneficiaries: site of death, place of care, and health care transitions in 2000, 2005, and 2009. JAMA..

[19] LaMantia MA, Scheunemann LP, Viera AJ, Busby-Whitehead J, Hanson LC (2010). Interventions to improve transitional care between nursing homes and hospitals: a systematic review. J Am Geriatr Soc.

[20] Intrator O, Zinn J, Mor V (2004). Nursing home characteristics and potentially preventable hospitalizations of long-stay residents. J Am Geriatr Soc.

[21] Grabowski DC, O'Malley AJ, Barhydt NR (2007). The costs and potential savings associated with nursing home hospitalizations. Health Aff (Millwood).

[22] Ouslander JG, Lamb G, Tappen R, Herndon L, Diaz S, Roos BA (2011). Interventions to reduce hospitalizations from nursing homes: evaluation of the INTERACT II collaborative quality improvement project. J Am Geriatr Soc.

[23] Ouslander JG, Lamb G, Perloe M, Givens JH, Kluge L, Rutland T (2010). Potentially avoidable hospitalizations of nursing home residents: frequency, causes, and costs: [see editorial comments by Drs. Jean F. Wyman and William R. Hazzard, pp 760-761]. J Am Geriatr Soc.

[24] Kramarow EA, Tejada-Vera B (2019). Dementia mortality in the United States, 2000–2017.

[25] Dunn A, Grosse SD, Zuvekas SH (2018). Adjusting health expenditures for inflation: a review of measures for health services research in the United States. Health Serv Res.

[26] Park RE (1966). Estimation with heteroscedastic error terms. Econometrica..

[27] Pregibon D (1980). Goodness of link tests for generalized linear models. J R Stat Soc: Ser C: Appl Stat.

[28] Graubard BI, Korn EL (1999). Predictive margins with survey data. Biometrics..

[29] Williams R (2012). Using the margins command to estimate and interpret adjusted preditions and marginal effects. Stata J.

[30] Park S, Roby DH, Pintor JK, Stimpson JP, Chen J, Ortega AN (2019). Insurance coverage and health care utilization among Asian youth before and after the affordable care act. Acad Pediatr.

[31] Park S, Stimpson JP, Pintor JK, Roby DH, McKenna RM, Chen J (2019). The effects of the affordable care act on health care access and utilization among Asian American subgroups. Med Care.

[32] Park S, Chen J, Roby DH, Ortega AN. Differences in health care expenditures among non-Latino whites and Asian subgroups vary along the distribution of the expenditures. Med Care Res Rev. 2019:1077558719874212. 10.1177/1077558719874212. Epub ahead of print. PMID:31524050. 10.1177/1077558719874212 31524050

[33] Park S, Chen J, Ma GX, Ortega AN (2019). Utilization of essential preventive health services among Asians after the implementation of the preventive services provisions of the affordable care act. Prev Med Rep.

[34] Husaini BA, Sherkat DE, Moonis M, Levine R, Holzer C, Cain VA (2003). Racial differences in the diagnosis of dementia and in its effects on the use and costs of health care services. Psychiatr Serv.

[35] Gilligan AM, Malone DC, Warholak TL, Armstrong EP (2013). Health disparities in cost of care in patients with Alzheimer's disease: an analysis across 4 state Medicaid populations. Am J Alzheimers Dis Other Dement.

[36] Yaffe K, Falvey C, Harris TB, Newman A, Satterfield S, Koster A (2013). Effect of socioeconomic disparities on incidence of dementia among biracial older adults: prospective study. BMJ..

[37] Mead H, Cartwright-Smith L, Jones K, Ramos C, Siegel B, Woods K. Racial and ethnic disparities in U.S. health care: a chartbook. The Commonwealth Fund; 2008.

[38] Kirby JB, Lau DT (2010). Community and individual race/ethnicity and home health care use among elderly persons in the United States. Health Serv Res.

[39] Burton L, Kasper J, Shore A, Cagney K, LaVeist T, Cubbin C (1995). The structure of informal care: are there differences by race?. Gerontologist..

[40] Dilworth-Anderson P, Williams IC, Gibson BE (2002). Issues of race, ethnicity, and culture in caregiving research: a 20-year review (1980-2000). Gerontologist..

[41] Zuckerman IH, Ryder PT, Simoni-Wastila L, Shaffer T, Sato M, Zhao L (2008). Racial and ethnic disparities in the treatment of dementia among Medicare beneficiaries. J Gerontol B Psychol Sci Soc Sci..

[42] Snowden LR (2001). Barriers to effective mental health services for African Americans. Ment Health Serv Res.

[43] Desai U, Kirson NY, Ye W, Mehta NR, Wen J, Andrews JS (2019). Trends in health service use and potentially avoidable hospitalizations before Alzheimer's disease diagnosis: a matched, retrospective study of US Medicare beneficiaries. Alzheimers Dement (Amst).

[44] Weuve J, Barnes LL, Mendes de Leon CF, Rajan KB, Beck T, Aggarwal NT (2018). Cognitive aging in Black and White Americans: cognition, cognitive decline, and incidence of Alzheimer disease dementia. Epidemiology..

[45] Luo H, Yu G, Wu B (2018). Self-reported cognitive impairment across racial/ethnic groups in the United States, National Health Interview Survey, 1997-2015. Prev Chronic Dis.

[46] Ellis RP, Hsu HE, Song C, Kuo TC, Martins B, Siracuse JJ (2020). Diagnostic category prevalence in 3 classification systems across the transition to the international classification of diseases, tenth revision, , Clinical Modification. JAMA Netw Open.

[47] Stewart CC, Lu CY, Yoon TK, Coleman KJ, Crawford PM, Lakoma MD (2019). Impact of ICD-10-CM Transition on Mental Health Diagnoses Recording. EGEMS (Wash DC).

[48] Yoon J, Chow A (2017). Comparing chronic condition rates using ICD-9 and ICD-10 in VA patients FY2014-2016. BMC Health Serv Res.

